# Septal Accessory Pathway: Anatomy, Causes for Difficulty, and an Approach to Ablation

**Published:** 2010-07-20

**Authors:** Paula G Macedo, Sandeep M Patel, Susan E Bisco, Samuel J Asirvatham

**Affiliations:** 1 Department of Internal Medicine, Mayo Clinic, Rochester, Minnesota; 2 Division of Cardiovascular Diseases, Mayo Clinic, Rochester, Minnesota; 3 Department of Pediatrics and Adolescent Medicine, Mayo Clinic, Rochester, Minnesota

**Keywords:** Septal, Accessory Pathway, Children, Complications, Electrophysiology

## Abstract

Accessory pathway (AP) ablation is one of the most satisfying invasive electrophysiology procedures associated with high success rates and relatively few complications.  Nevertheless, when APs are found on the cardiac septum, ablative procedures become complex, and unique pitfalls need to be avoided.

These difficulties with septal ablation are magnified in the pediatric population.  The relatively small heart, rapid nodal conduction, and proximity of the arterial system specifically complicate septal ablation in children.  The electrophysiologist must use every tool in his or her armamentarium, including exact delineation of pathway location, identification of pathway potentials, detection of the presence of pathway slant, etc.  In addition, an exact knowledge of the complex anatomy of the cardiac septum, including the posteroseptal space, the aortic cusp region, and the proximity of the AV conduction system and coronary vessels, becomes mandatory.

In this review, we describe the developmental anatomy and regional anatomy of septal accessory pathways.  We then discuss approaches to map specific to pathways in particularly problematic regions at or near the septum, including venous and aortic cusp related accessory pathways.

## Introduction

**Figure 1:**
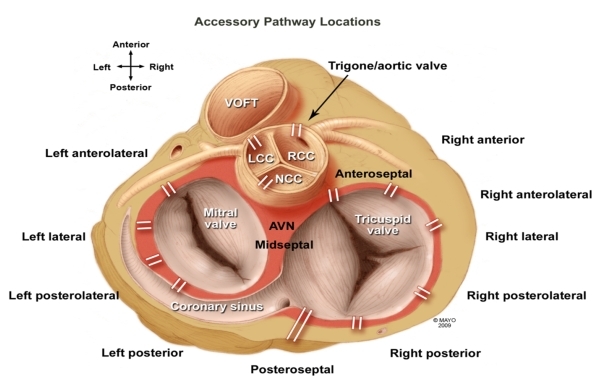
Atrial ventricular accessory pathways may occur anywhere along the atrial ventricular annulus.  Note the posteroseptal region is not a part of the true septum.  Note also the position of the aortic annulus interspersed between the right and left anteroseptal regions.  Midseptal pathways are among the most difficult to ablate because of the proximity of the AV node. VOFT  = ventricular outflow tract; LCC = left coronary cusp; RCC = right coronary cusp; NCC = noncoronary cusp; AVN = AV node

Accessory atrioventricular pathway ablation has become a common occurrence in the field of electrophysiology. Septal accessory pathways (APs) are, however, known to be difficult to ablate as compared to those in other locations [1]. Because of the difficulty, it becomes clinically relevant for the practicing electrophysiologist to understand the anatomy, development, and practical methods for performing successful ablations, especially in the posteroseptal region. The posteroseptal region of the heart is probably the most complex area among those that harbor AV accessory fibers.  There is no sharp true demarcation between this region and its surrounding regions including the midseptal, left posterior paraseptal, and right posterior paraseptal (Figure 1) [2]. Furthermore, a higher recurrence rate and potential for AV block with ablation have been reported in patients with septal APs [1]. Therefore, it is imperative to understand the anatomy and development of this area in order to successfully and safely ablate pathways in this location.

## Anatomy

Previous surgical experience has divided the septum into anterior and posterior divisions.  The point of division is made up of the annuli of the two AV valves, the aortic annulus, the membranous ventricular septum, and atrial septum, i.e. the right fibrous trigone [1]. The posterior AV septum is bound by the posteromedial aspects of the left and right atrium, the superior process of the posterior confluences of the left and right ventricular walls, and the epicardium over the crux of the heart [1]. The right atrial boundary also contains the orifice of the coronary sinus (CS) [1]. The smaller anterior portion of the AV septum is bounded posteriorly by the right fibrous trigone; laterally by the epicardial side of the right atrium and the annulus fibrosus; medially and anteriorly by the epicardium that is reflected from the aortic root and by the infundibulum to the right atrium; and inferiorly by the muscular ventricular septum and the right ventricular infundibulum.

**Figure 2:**
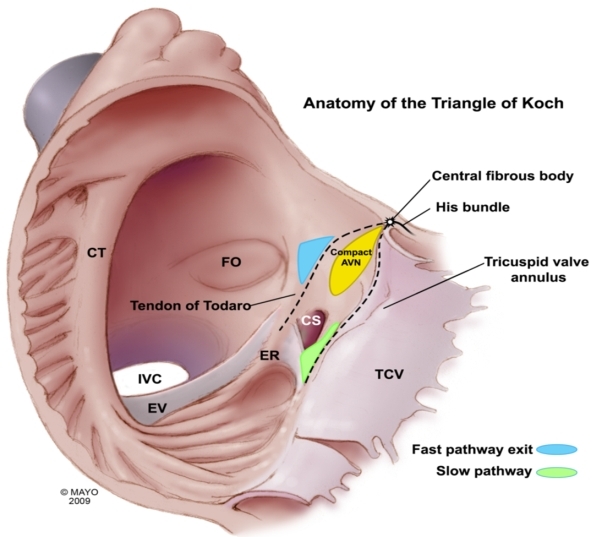
The true interatrial septum near the tricuspid annulus consists of the anterior and midseptal regions.  Midseptal pathways are inserted into the triangle of Koch region, close to the compact AV node.  Accurate assessment of pathway slant and ablation preferably of the ventricular insertion site along with the use of cryoenergy may all be required to minimize the risk of AV block when ablating pathways in this region. CT = cristoterminalis ; FO = Forman ovale; EV = Eustachian valve; IVC = inferior vena cava; TCV = tricuspid valve; CS = coronary sinus; ER = Eustachian Ridge; AVN = AV node

As further research into the septal regions has advanced, previous clinical designations of the septal regions are not regarded as entirely accurate. Dean et al have reported that the mid septum is the only true muscular septal area between the offset attachments of the mitral and tricuspid valves, while the previously named anterior and posterior septal areas are those regions that are anterior and posterior to the true septum [1]. This true septum corresponds roughly to the location of the triangle of Koch. The triangle is of great value to the electrophysiologist because it marks the approximate location of the AV node and bundle of His. Bounded by the tendon of Todaro (linear tissue that runs from the central fibrous body to the Eustachian ridge), the tricuspid annulus, and the orifice of the coronary sinus, the AV node is situated at the apex of this triangle. The APs that occur within the septum typically occur circumferentially along the annuli of the mitral and tricuspid valves (Figure 2) [1].

## Development and Embryology

 Atrial septation during cardiac development is an extremely complex process. The concept of converting a single myocardial tube into four cardiac chambers involves multiple mechanisms, including looping, the formation of extra cardiac mesenchyme, the segmental transformation of endocardial endothelium into valvuloseptal (cushion) mesenchyme, and finally the remodeling of the myocardial epithelium into muscular partitions [3]. It is important to note that the septation process is reflected in the position of the primitive conduction system. At the level of the atrium, the sinus venosus and sinoatrial transition to form the sinoatrial node [4]. During this period of development, multiple accessory pathways run through the areas that are to become the right and left cardinal veins, pulmonary veins, coronary sinus, and right atrioventricular ring [4]. However, in the mature adult heart, this primitive conduction tissue is not recognizable anymore, and usually undergoes electrical insulation via formation of the annulus fibrosus.  When insulation does not occur appropriately, the result is the persistence of APs [5,6]. Most APs are found within the parietal atrioventricular junctional areas, including the paraseptal region [5]. These APs consist mostly of myocardium and are rarely specialized cells. Since they are derived from the same precursor, many of their characteristics are similar to normal cardiac myocytes [5]. However, refractory periods during cardiac repolarization differ in length and provide the ability for faster conduction or faster reexcitation. AP origin on the atrial aspect is thick while insertion on the ventricular side is through thin branches that are electrically connected on both sides by gap junctions [5]. Accessory atrioventricular pathways are thought to develop secondary to disruption of the electrical insulation between the atria and the ventricles. The cushion tissue forms the internal scaffolding which will become the valve leaflets and contribute to the formation of the annulus fibrosis for electrical insulation [5,6]. Recent studies in this area from Gittenberger-de Groot et al have shown the importance of the epicardially-derived cells (EDCs) in the formation of this fibrous tissue. By inhibiting migration of EDCs in quail embryos, Gittenberger-de Groot et al observed the persistence of broad bundles of accessory atrioventricular canal myocardial connections that resulted in ventricular pre-excitation  [7].

## Posteroseptal Pathways

### Anatomy and Electrocardiography

In many studies, ablation of the posteroseptal region has been identified as being more difficult than for pathways located in other areas [8,9]. Of note, ablation in this area has been associated with longer procedure times, longer radiation exposure, and the need for more radiofrequency (RF) pulses to accomplish successful results [9]. Additionally, ablation of these APs has been known to require RF energy within the CS, including the middle cardiac veins and diverticulum; therefore, it is a special importance to understand the complexity of this region.

The posteroseptal region is specifically located between the posterosuperior process of the left ventricle and inferior wall of the right atrium [2], i.e. the crux. The crux anatomically corresponds to the area where the four cardiac chambers reach their maximum proximity posteriorly. The posterior septum is bounded by three distinct areas: 1) midseptum (anterior and superior borders); 2) right posterior paraseptal (right lateral border); and 3) left posterior paraseptal (left lateral border) [2].

The posteroseptal region can be anatomically divided into right and left regions that correspond to the annuli of the tricuspid and mitral valves, respectively. A posteroseptal AP is defined as one located posterior or apical to the orifice of the CS.  Surface EKGs typically show the following preexcitation features: 1) R/S ratio > 1 in V2; 2) negative delta wave in III; and 3) R/S ratio < 1 in III [10,11]. Furthermore, nearly all posteroseptal pathways have negative P waves in the three inferior leads and positive P waves in lead I. Studies have shown difficulty in discriminating left- from right-sided posteroseptal pathways [12]; however, it has been suggested that a positive delta wave in V1 can further differentiate a left-sided from a right-sided posteroseptal pathway [2,12]. Additionally, the frontal axis of the delta wave in a right-sided pathway was -30 to 50, and -60 or greater in left-sided pathways [2].

### Mapping and Ablation

The comments presented below may be applied for mapping and ablation anywhere on the cardiac septum but are perhaps best exemplified when dealing with the posteroseptal space. Specific differences in other septal locations are presented in the appropriate sections that follow.

#### Exact Mapping

**Figure 3:**
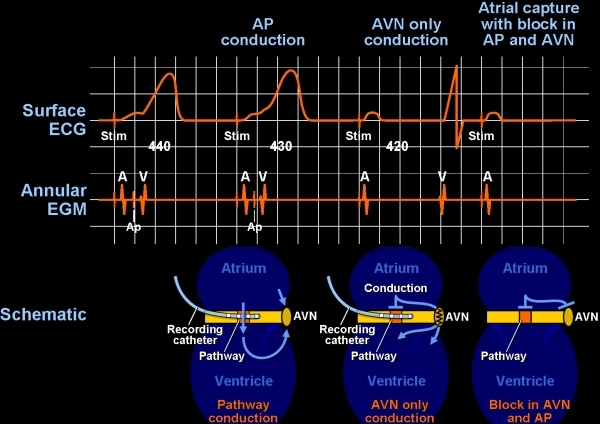
Accurate diagnosis of a candidate potential as an accessory pathway potential is important for any accessory pathway ablation, but it is critical for safe ablation of septal pathways.  The suspicious potential in this patient with an antegrade conducting pathway is noted between the atrial (A) and ventricular (B) electrograms.  The operator must distinguish the AP potential from a fragmented portion of the atrium or ventricular electrograms.  The most important, however, is to make sure this is not part of the atrial electrogram, since targeting such an electrogram will be meaningless.  Pacing at a more rapid rate causes block in the accessory pathway and subsequently block in the AV node, as well.  The atrial electrogram is seen to be easily dissociated from the accessory pathway potential (Ap) thus ensuring that it was not a fragmented portion of the V.  AP = accessory pathway; EGM = electrogram; ECG = electrocardiogram;

**Figure 4:**
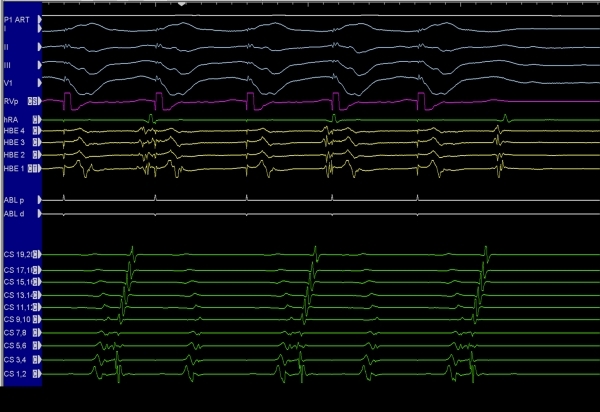
Ventricular pacing in a patient with a left-sided accessory pathway, and having failed multiple prior ablation attempts.  Fragmented electrograms are seen on the coronary sinus electrode (CS5, 6) etc.  Ventricular pacing results in V-A block.  The first two components of the complex signal cannot be dissociated from the ventricular electrogram, and thus likely represent complex ventricular signals.  Atrial extrastimuli can be given during ventricular pacing to further distinguish the last two components of the complex electrograms as to whether they represent fragmented atrial electrograms or pathway potential and an atrial electrogram.

Although precise mapping is desirable for pathway ablation anywhere in the heart, it is essential when targeting APs on the septum.  In general, electrophysiologists use various criteria for targeting pathway locations for energy delivery [13,14]. However, some of these approaches are best avoided when ablating on the septum:

1. *Shortest AV interval*. Caution should be used when solely targeting the shortest AV (atrial pacing) or V-A interval (ventricular pacing). Depending on the pacing site, the V-A interval may be 0 ms at sites far away from the actual AP. In those instances, the short interval is simply a reflection of simultaneous activation of the atrium and ventricle at that time (pseudo interval) picked up by the electrode on the annulus. The situation can be clarified by pacing close to the suspected AP site, but this is not straightforward to do with septal pathways  [13,15,16].
2. *Earliest ventricular activation during atrial pacing or earliest atrial activation during ventricular pacing/orthodromic reciprocating tachycardia*. Although this technique can be applied here again on the septum, it is not ideal.  For example, the earliest atrial activation for a downward slanted posteroseptal pathway may be very close to the compact AV node, and safe ablation could have been done more inferiorly on the annulus itself [17-19].
3. *The pathway potential*. In general, discerning a pathway potential and targeting this signal for ablation is a superior method, but this is most acutely valid with septal pathways. Pacing maneuvers, so as to separate the candidate signal (possible pathway potential) from the atrial electrogram, ventricular electrogram, and in some instances His bundle electrogram needs to be done to validate the signal. Once certain of the nature of the signal and after establishing adequate contact, the pathway potential can be targeted for energy delivery (Figure 3, Figure 4)  [20-24].

Another concept that should be thoroughly understood when attempting ablation on the septum is that of pathway slant. This basic concept has been well-illustrated by Gonzales et al [25,26]. Briefly, pacing on either side of the AP results in (for example) a retrograde conducting left-sided AP. Pacing on either side of the ventricular insertion will result in variation in the local V-A interval measured at an electrode on the annulus. When pacing along the slant (closer to the ventricular insertion), the V-A interval is shorter and vice versa. This technique, however, needs to be modified when assessing supero-inferior slant in the septum with pacing sites chosen superior to and inferior to the insertion site of the pathway. The value of knowing this technique well is that electrophysiologists may then chose to ablate along the slant in such a way as to avoid potential collateral damage. For instance, if a posteroseptal pathway is slanted so that the atrial insertion is more superior and the ventricular insertion is more inferior, ablation can be targeted at the ventricular insertion site (more posteriorly) to minimize the risk of AV nodal damage [14,27,28].

**Figure 5:**
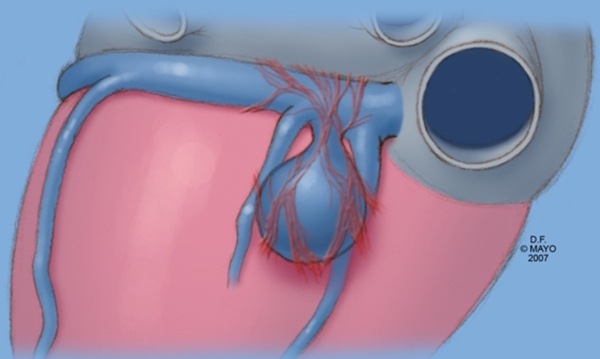
Illustration of myocardial fibers on a coronary sinus diverticulum.  In this instance, the ostium of the diverticulum is located between the middle cardiac vein and a posterior ventricular vein.  Note that the myocardial fibers essentially are a continuation of the usual fibers found in the proximal coronary sinus.  When these fibers connect to both the atrial myocardium and ventricular myocardium, a complex epicardial accessory pathway is created.  One approach to ablation of these types of pathways involves circumferential ablation at the mouth of the diverticulum.

**Figure 6:**
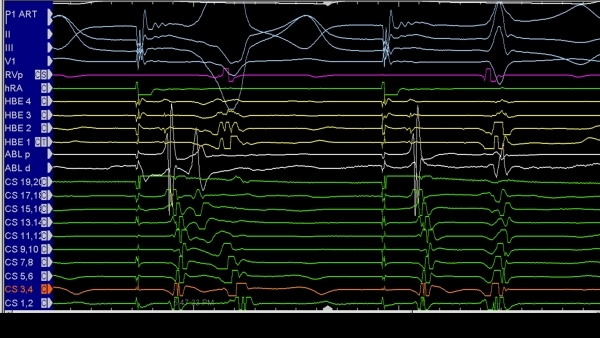
Intracardiac electrocardiograms during encircling type ablation done at the ostium of a coronary sinus diverticulum.  The last portion of the circumference is being ablated (ABLT and ABLD ablation catheters proximal and distal electrodes) with block in accessory pathway conduction resulting.  Tachycardia was no longer inducible in this patient. CS = coronary sinus; HBE = His bundle electrogram; RV = right ventricle, HRA = High right atrial; ECG leads I, II, III, and V1

While the principles described above (along with the accompanying figures and explanations) elucidate the salient concepts needed for careful ablation of any septal pathway, there are certain peculiar issues that arise with posteroseptal pathway ablation, especially for epicardial pathways involving the coronary sinus myocardium (Figure 5, Figure 6). The most important of these specific issues is detailed below where we discuss the potential complication of coronary artery injury followed by a brief description of techniques to avoid this complication.

### Complications of Ablation - Coronary Artery Injury

RF catheter ablation can lead to rare but serious consequences. Coronary artery injury after ablation may present acutely or several weeks after an ablation procedure. Stenosis of the coronary arteries immediately and during long-term follow-up after ablation has been described in animal models [29-31]. However, the overall incidence of coronary injuries during ablation procedures is extremely low [32]. In one large retrospective study, clinically evident coronary injury incidence is estimated to be 0.06% - 0.1% in adults [33,34]. This low risk is surprising given the close proximity of the coronary arteries to common sites of ablation and may be due to under-recognition and underreporting [32].

Patients typically present with chest discomfort during and after ablation, likely secondary to pericardial irritation. Several authors have attributed this presentation to coronary vasospasm because of reversible ST segment elevation but with normal angiography [32,33,35,36]. The mechanism for this is unclear; however, spasm is thought to occur from thermal energy transmitted during ablation [37]. Conversely, in children coronary angiograms taken after ablation have shown stenosis of coronary arteries occurring in as little as 30 minutes from the conclusion of the procedure [36]. In animal models, the application of RF energy directly on a coronary artery causes acute edema with wall thickening and luminal narrowing [209]. In a matter of days, medial necrosis with loss of intimal and elastic tissue with the subsequent development of severe intimal hyperplasia develops [29,38]. In the setting of delayed coronary injury, the presumption is that in addition to intimal hyperplasia, ablation damages the arterial wall and endothelium, which provides a nidus for progressive thrombus formation weeks later [32].

Multiple case reports have documented that ablation of a posteroseptal pathway has led to injury of the coronary arteries [20,39,42]. Again, the complex anatomy of this region and the pathways that traverse it may provide the insight to these observations. In children, a study of postmortem pediatric cardiac histology has demonstrated that the distance between the endocardium at the AV groove and the coronary arteries shown to be shortest within the CS and in the posteroseptal region, generally less than 4 cm [43]. In adults, a significant coronary artery is found within 2 mm of the best ablation site in the CS or its branches in nearly two-thirds of patients, and ablation at this site causes coronary stenosis in two-thirds of patients [29]. Furthermore, Hasdemir et al have examined the anatomic relationship of the coronary arteries to the tricuspid and mitral annulus and finds that the right coronary artery (RCA) is < 5 mm from the cavotricuspid isthmus in 8% of patients, and the left circumflex artery is ≤ 2 mm from the lateral mitral annulus in 24 % of patients [44]. The CS and its branches are also in close proximity to the distal circumflex and the posterolateral branches of the RCA, as well [31]. These anatomic relationships explain why the usual ablation of posteroseptal pathways, specifically right-sided, usually results in damage to the distal RCA, as previously reported in adults.

### Avoiding Collateral Damage

#### Right coronary artery occlusion

**Figure 7:**
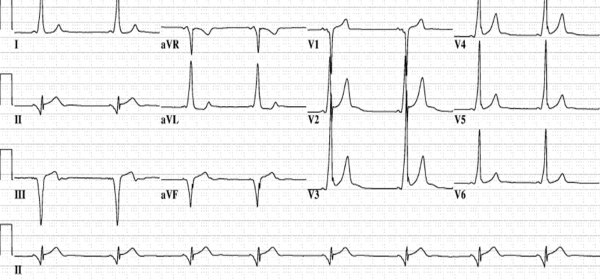
Classic electrocardiographic pattern in a patient with a posteroseptal accessory pathway.  The delta wave transition occurs between V1 and lead II.22  The delta wave is negative in II, III, and AVF, placing the pathway in the posteroseptal region.  However, the pathway can be further localized to the middle cardiac vein or posterior vein, since the delta wave is immediately negative in lead II.  (See text for details)

As mentioned before, ablation attempts into the middle cardiac vein and/or the coronary sinus can result in damage to a branch of the RCA (Figure 7). There are some reports describing coronary occlusion following RF deliveries in that region, prompting urgent angioplasty [39-42]. The acute injury can be secondary to spasm, edema, thrombosis with complete obstruction, coronary embolization, or direct intimal trauma. Chronic occlusion can also occur and is secondary to progressive scarring.

**Figure 8:**
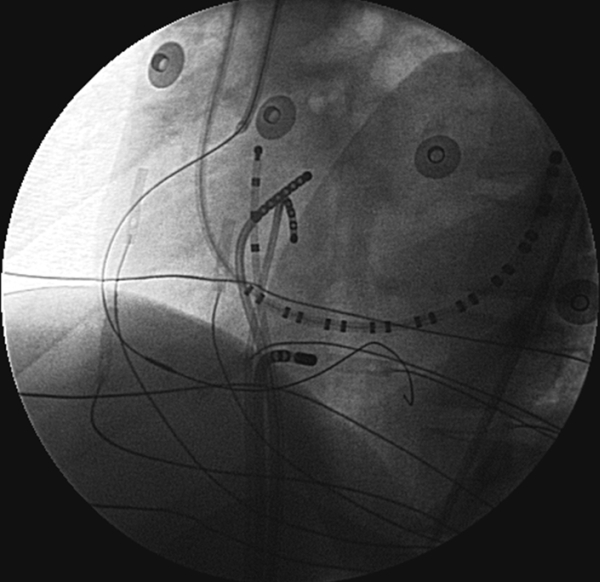
Left anterior oblique fluoroscopic image showing an ablation catheter placed near the ostium of the middle cardiac vein.  A guiding sheath has engaged the right coronary artery and an intravascular ultrasound probe placed into the arterial system.  The probe can be moved so as to image the relevant heart rate when applying radiofrequency energy within a proximate vein.

With the intention of preventing this serious complication, anticipated angiography should be done. In cases that the artery is more than 5 mm away from the ablation site, energy can be safely delivered [45]. If the artery is closer, an intravascular ultrasound catheter should be placed to monitor for damage. When it is extremely close, cryoenergy can be used instead of RF. Cryoablation is safer and yields good acute success rates for AP ablation [46-48], although it is also associated with higher recurrences (Figure 8).

## PJRT - Permanent Form of Junctional Reciprocating Tachycardia

These accessory pathways are often found near or on the septum [49] and can be difficult to target for ablation. The characteristic of these APs is decremental conduction with long conduction times. As a result, distinguishing this arrhythmia (PJRT) from AV node reentry or atrial tachycardia may not be straightforward. Typical techniques, including parahisian pacing, PVCs to preexcited tachycardia, etc., may not be revealing because of the decremental conduction these pathways show. However, the electrophysiologist can use the decremental nature of these pathways as an advantage when trying to elucidate the tachycardia mechanism. For instance, PVCs may routinely post-excite the tachycardia or terminate the tachycardia, despite being delivered at a time of His bundle refractoriness. Moreover, because of the long ventriculoatrial conduction time, AP signals can in general be easier to see and establish in PJRT compared to regular AP-related tachycardias [50-52].

## Midseptal Pathways

Ablation at the midseptal region septum is particularly risky because of the intricate conduction system. Inadvertent second- and third-degree AV block has been reported, especially in children, because of the relative proximity of the AV node to the posteroseptal region [34,45,53]. In the Pediatric Radiofrequency Ablation Registry reported in 1996, ablation of midseptal APs was more frequently associated with accidental AV block (10.4 %) in comparison to other septal locations (anteroseptal 2.7 % and posteroseptal 1 %) [54]. One out of the three described deaths was secondary to complete AV block and subsequent cardiogenic shock while attempting ablation of a mid-septal AP  [53]. More recently, also in the pediatric group, it was demonstrated that high-grade AV block occurred in 10% of mid-septal AP ablations and in 5% of anteroseptal AP ablations [55]. Although in most of those cases the high probability of block was anticipated, the ablation was necessary due to refractoriness to medication and highly symptomatic arrhythmia. Thus, although with anteroseptal pathways we may see a His bundle electrogram on the catheter, the greatest care must be used when ablating on the mid septum because of the anatomic location of the compact AV node in this region. RF energy should never be applied as first line therapy on the midseptal region, and attempts with cryoablation first is generally prudent. Further, because the AV node is an atrial structure, ablation energy delivery should be exactly at the annulus or at ventricular locations when performed near the midseptal region, and it is critical to define accurately the location of the pathway prior to delivering either kind of energy.

## Anteroseptal Pathways

**Figure 9:**
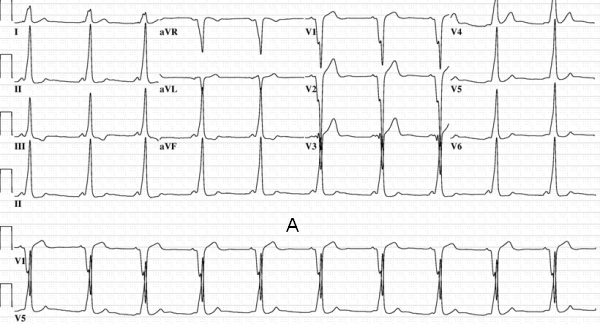

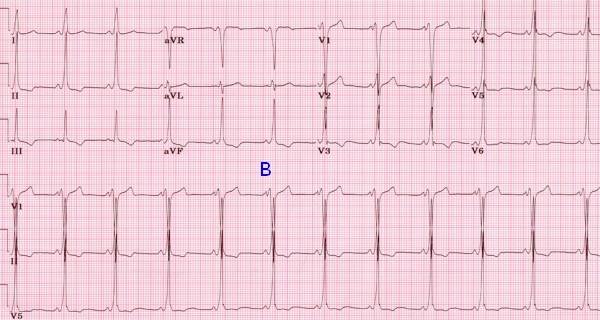
Panel A: Characteristic electrocardiogram in a patient with an anteroseptal pathway. Strongly positive delta waves in leads II, III, and AVF are noted.  The delta wave is isoelectric/negative in lead V1. Successful ablation was performed at a location on the anteroseptal annulus where a His bundle electrogram was being recorded. Ablation was very anterior, and thus not in the vicinity of the compact AV node.  Further, the ablation was performed targeting the ventricular insertion, as well. Panel B: In contrast, this preexcited electrocardiogram was obtained in a patient with successful ablation on the supravalvar aortic cuspal region.  Although the delta waves are positive in leads II, III, and AVF, note the slightly positive delta wave in lead V1 suggesting that the pathway is not on the true septum (see text for details).

An anteroseptal pathway is a less common cause of AV re-entrant tachycardia and accounts for 9% of all encountered APs [54,55]. The penetrating bundle of His is a well-insulated structure since it carries a sleeve of fibrous annular tissue for variable distances into the ventricle, losing this insulation as the right bundle exits to the myocardium. As a result, ablation is generally better tolerated on the His bundle itself compared to the fragile and easily ablated compact AV node. Anatomically, since the His bundle is a ventricular and anterior structure, whereas the compact AV node is a midseptal and atrial structure, more caution is needed whenever ablating lower than and more atrially than the anteroseptal region. As with midseptal pathways, it is generally preferable to attempt ablation with cryoenergy after the pathway has been mapped in this region (Figure 9) [46,55].

One variant of anteroseptal pathways are pathways that involve aortic cuspal ablations. 

## Aortic Cuspal Ablation

**Figure 10:**
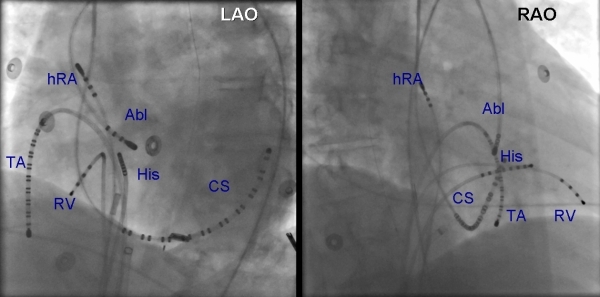
Left anterior oblique (LAO) and right anterior oblique (RAO) projection of a patient with successful ablation of an accessory pathway in the supravalvar aortic cusp region.  ABL ablation catheter in the left coronary cusp/noncoronary cusp junction.  HRA = high right atrium; TA = tricuspid annulus catheter; CS = coronary sinus catheter; RV = right ventricular catheter.

There are rare situations in which an anteroseptal AP could be ablated from the noncoronary aortic cusp [56,-58]. The substrate for ablation is still unclear, but myocardium potentials have been encountered in the supravalvar area [59]. Therefore, bridges across the aortic valve connecting the atrial and ventricular myocardium could constitute the AP. Another possibility is that they transverse the fibrous trigone in this region of the heart, but the aortic cusps would still be the better approach for ablation [59]. In this context, it is very important to remember mapping the aortic valve region whenever attempting ablation of an anteroseptal pathway. Correct identification of the pathway potential would allow successful ablation of the substrate while avoiding collateral AV conduction damage (Figure 10) [58,60].

## Uncommon Septal Accesory Pathways

**Figure 11:**
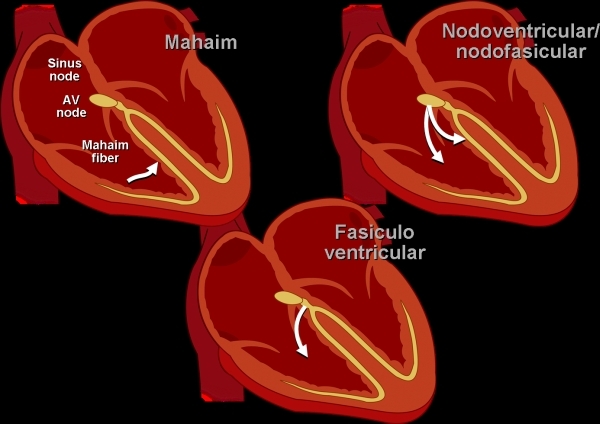
Figurative illustration of unusual pathways.  Mahaim fibers are true atrial ventricular connection, however, the connection occurs through an AV node and Hispurkenje-like structure (Mahaim fiber).  Fasciculoventricular tracts are not true accessory pathways, but rather “breaches in the insulation” of the proximal His bundle and right bundle region such that early activation of the basal portion of the right ventricle occurs with a short H-V interval and a suggestion of preexcitation.  Nodal ventricular and nodal fascicular fibers are rare accessory pathways that may participate in tachycardia (see text for details).

In addition to true atrial ventricular accessory pathways that happen to be located on the septum or the aortic valve region described above, certain pathway variants may present as potential septal accessory pathways:

1. Fasciculoventricular tracts [61] are not true atrial ventricular bypass tracts. Rather, they represent early excitation of the ventricle as a result of the absence of the usual fibrous insulating coat of the His bundle and right bundle branch. This results in a short H-V interval and the presence of preexcitation on the electrocardiogram. However, since conduction is still through the AV node, the H-V interval will not shorten nor become negative with decremental atrial pacing, and the extent of preexcitation does not change with either the atrial pacing site or with concurrent use of adenosine or with decremental pacing. Fasciculoventricular tracts are benign and not associated with any reentrant arrhythmia and should not be targeted for ablation.
2. Another rare variant involves the so-called nodal fascicular or nodal ventricular tracts (Figure 11). These tracts may be associated with tachycardia, and earliest atrial activation is found in the atria exactly at the site of early atrial activation with retrograde nodal conduction [62,63]. Ablation can be successfully performed without AV nodal damage targeting the nodal fascicular or nodal ventricular fiber or in some instances in the region of the CS ostium [64,65]. True atrial ventricular tracts with decremental conduction (Mahaim) fibers are generally thought not to occur on the septum; however, some nodal fascicular tracts, especially when ablated in the posteroseptal region, may have represented true AV bypass tracts involving a small portion of the atrial myocardium ventricular to the tendon of Todaro. Although very rare, these variants should be kept in mind when approaching patients with septal accessory bypass tracts.

## Summary

Although mapping and ablation of accessory bypass tracts in patients with symptomatic tachycardias is a highly rewarding, successful, and safe procedure, septal pathways remain challenging. Posterior septal pathways, especially when epicardial and involving the coronary sinus myocardium or middle cardiac venous myocardium can be very difficult to ablate because of the proximity of the arterial system. Accurate knowledge of the anatomy involved, use of cryoenergy, adjunctive coronary angiography and intravenous ultrasound, and careful mapping within the veins or venous diverticula may lead to success.

Midseptal pathways are particularly likely to be complicated with damage to the compact AV node.  In patients with anteroseptal pathways, careful delineation of the pathway potential with energy delivery sometimes involving the aortic cusps should be considered.
